# The Physical Layer Security Experiments of Cooperative Communication System with Different Relay Behaviors

**DOI:** 10.3390/s17040781

**Published:** 2017-04-06

**Authors:** Yishan Su, Guangyao Han, Xiaomei Fu, Naishen Xu, Zhigang Jin

**Affiliations:** 1 School of Marine Science and Technology, Tianjin University, 300072 Tianjin, China; yishan.su@tju.edu.cn; 2 School of Electrical Automation and Information Engineering, Tianjin University, 300072 Tianjin, China; hanguangyao@tju.edu.cn (G.H.); xunaishen@126.com (N.X.); zgjin@tju.edu.cn (Z.J.)

**Keywords:** physical layer security, different relay behavior, experiment

## Abstract

Physical layer security is an attractive security mechanism, which exploits the randomness characteristics of wireless transmission channel to achieve security. However, it is hampered by the limitation of the channel condition that the main channel must be better than the eavesdropper channel. To alleviate the limitation, cooperative communication is introduced. Few studies have investigated the physical layer security of the relay transmission model. In this paper, we performed some experiments to evaluate the physical layer security of a cooperative communication system, with a relay operating in decode-and-forward (DF) cooperative mode, selfish and malicious behavior in real non-ideal transmission environment. Security performance is evaluated in terms of the probability of non-zero secrecy capacity. Experiments showed some different results compared to theoretical simulation: (1) to achieve the maximum secrecy capacity, the optimal relay power according to the experiments result is larger than that of ideal theoretical results under both cooperative and selfish behavior relay; (2) the relay in malicious behavior who forwards noise to deteriorate the main channel may deteriorate the eavesdropper channel more seriously than the main channel; (3) the optimal relay positions under cooperative and selfish behavior relay cases are both located near the destination because of non-ideal transmission.

## 1. Introduction

When it comes to the secure transmission of information, cryptographic algorithms are traditionally employed as the security technology to achieve information confidentiality [1]. Information encryption usually involves the use of a key to encrypt and decrypt the messages. Unlike conventional wired communication that uses wired media (optical cable, copper, etc.) for data transmission, the broadcast nature of wireless channels allows an illegal receiver to wiretap legal messages easily during the transmission from the source to the destination. Moreover, with the development of cryptography, new algorithms and methods to decipher traditionally encrypted messages have been developed, which means old encryption methods are faced with higher risk of being cracked. Cryptographic methods face numerous risks due to the rapid development of the computing capabilities of eavesdroppers [2]. Besides, complex cryptographic algorithms require devices capable of dealing with heavy computing loads, leading to high power and resource costs. The physical layer security adopts the physical characteristics of wireless channels to guarantee its security through coding and signal processing, in which confidential message can be decoded only by their legitimate receiver and avoid complex computing. In this way, physical layer security has been proposed to safeguard wireless transmission, for instance, 5G wireless communication networks and heterogeneous cellular networks [3,4].

Physical layer security was first introduced by Wyner [5], who adapted Shannon’s concepts of entropy and equivocation [6] to investigate the physical layer security. The achieved maximum transmission rate of confidential message communication is defined by Wyner as the secrecy capacity. However, the feasibility of physical layer security is hampered by the limitation when the channel between the source and the destination (the main channel) is worse than that between the source and the eavesdropper (the wiretap channel). An effective way to improve the main channel condition is cooperative communication with a relay’s help [7].

Cooperative relays can enhance the secrecy capacity of wireless communications by exploiting decode and forward (DF), amplify and forward (AF) [8]. In the DF and AF cooperative strategies, the relay utilizes its overheard information to reinforce the transmitting signal of the source [9,10,11]. In [12,13,14], the physical layer security is improved by the advantages of large scale MIMO arrays of relays with AF and DF strategies. In [15,16], the optimal transmission with maximized secrecy rate under energy harvesting requirements is investigated, where the secrecy capacity is sensitive to the position among the nodes. It was found that the distance from the source to the relay has an important influence on secrecy capacity. Therefore, to find the optimal relay location is an important requirement to achieve maximum secrecy capacity [17]. In this paper, we investigated a cooperative communication system with one source, one relay, one destination and one eavesdropper with experiments for further discussion.

In practical applications, relays may not always select a cooperative strategy because of its own benefits. Some research has investigated the secrecy capacity with untrusted relays. In [18,19] the authors discussed the case of untrusted relays with both a cooperator and an eavesdropper. The secrecy capacity in a selfish behavior relay scheme in which the relay wants to save its own resources [20,21] was also discussed.

Theoretical analysis usually guides the real implementation with simplified or ideal physical factors, while applying them in practice may lead to different results. The authors in [22] firstly analyzed the physical layer security with the probability of a non-zero secrecy capacity ($P\left(C_{S}>0\right)$) of a wiretap channel by experimental measurements. It was found that the shadowing deviation can influence the security, which was seldom considered by theoretical analysis. The results from [23] can only be applied in a point-to-point network scenario. To investigate the physical layer security of multiple point relay transmission models, in this paper, we establish a cooperative communication testbed based on a software-defined radio (SDR) platform [24]. Based on this platform, we investigate the physical layer security with different relay behavior (cooperation, selfishness, maliciousness) with DF scheme in real non-ideal wireless transmission environment. The $P(C_{S}>0)$ was calculated to evaluate physical layer security in an actual outdoor environment. We obtain some different results which is ignored by theoretical or simulation analysis.

The paper is structured as follows: Section 2 introduces the system model and different relays’ behavior; Section 3 presents experimental results of bit error rate (BER) in a system with different relay behavior; Section 4 discusses the security performance under different relay behavior; finally, Section 5 presents our conclusions. The symbols used in this paper are described in Table 1.

## 2. System Description

### 2.1. System Model

**Figure 1:**
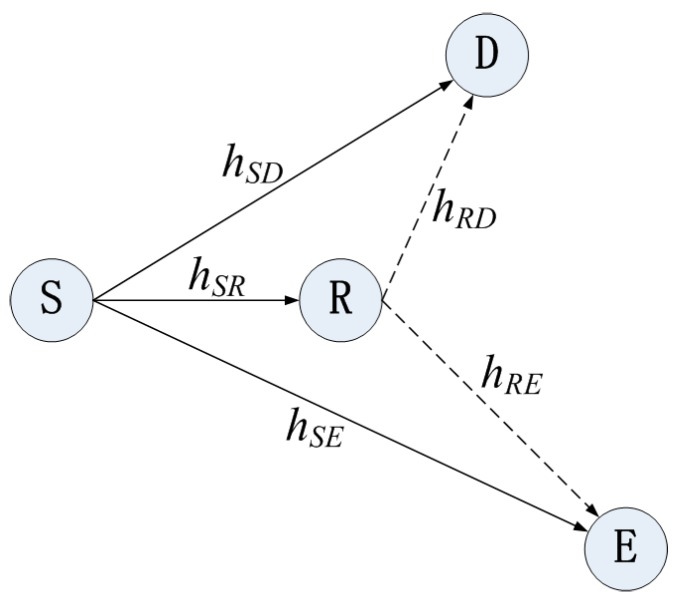
Relay transmission model with one eavesdropper, where $h_{SD},\;h_{SR},\;h_{RD}$ denote the channel fading coefficients from S to D, R, and from R to D. They are modeled as follows: $h_{SD}\sim CN\left(0,\sigma_{SD}^{2}\right)$, $h_{SR}\sim CN\left(0,\sigma_{SR}^{2}\right)$, $h_{RD}\sim CN\left(0,\sigma_{RD}^{2}\right)$, with $\sigma_{SD}^{2}=E\left\{{\left|h_{SD}\right|}^{2}\right\}$, $\sigma_{SR}^{2}=E\left\{{\left|h_{SR}\right|}^{2}\right\}$, $\sigma_{RD}^{2}=E\left\{{\left|h_{RD}\right|}^{2}\right\}$.

A relay transmission system model consists of a source (S), a destination (D), a relay (R) and an eavesdropper (E), as depicted in Figure 1. S wants to communicate with D in the presence of E. The S broadcasts signals with average power $P_{S}$. R receives signals from S with DF strategy.

### 2.2. Different Behaviors of Relay

In this paper, three types of relay behaviors are discussed:

1. Cooperative Behavior: The relay decodes the received signal and forwards it to the destination.
2. Selfish Behavior: The relay refuses to cooperate with probability $P_{off}$ [14], while selecting to cooperate with probability $P_{on}\;\left(P_{on}=1-P_{off}\right)$.
3. Malicious Behavior: The relay forwards random noise instead of the received signals of the source.

### 2.3. The Probability of Non-Zero Secrecy Capacity with Different Behavior

**Figure 2:**
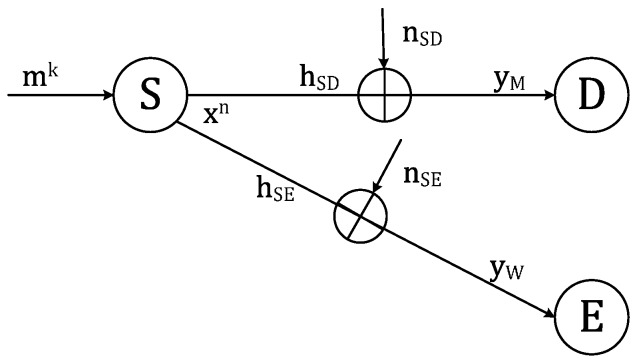
Wiretap communication model without relay.

To investigate the physical layer security in experiment, we adopted the theories and equations in [8] with the wiretap communication model without relay, as shown in Figure 2. Then, we investigate the scenario of a relay cooperative case. Suppose a *k*-length message $m^{k}$ sent from source is coded to n-length codeword $x^{n}(x^{n}=\left[x\left(1\right),x\left(2\right),\ldots ,x\left(n\right)\right]$), chosen from codebook $X^{n}\left(x^{n}\in X^{n}\right)$, the main channel and the wiretap channel output is described as:

(1) $$y_{M}\left(i\right)=h_{SD}\left(i\right)x\left(i\right)+n_{SD},$$

(2) $$y_{W}\left(i\right)=h_{SE}\left(i\right)x\left(i\right)+n_{SE},$$

where $h_{SD},h_{SE}$ denote the channel fading coefficients of the main channel and wiretap channel respectively with complex Gaussian distribution. Notations $n_{SD},\;n_{SE}$ denote the mean circularly symmetric complex Gaussian noise in the main channel and wiretap channel respectively, and $n_{SD}\sim CN\left(0,N_{M}\right)$, $n_{SE}\sim CN\left(0,N_{W}\right)$.

The channel power is limited by average transmit signal power *P*:

(3) $$\frac{1}{n}\overset{n}{\underset{i=1}{\sum }}E\left[{\left|x\left(i\right)\right|}^{2}\right]\leq P.$$

The average *SNR* of the main channel is given by:

(4) $$\gamma_{M}=P\times E\left[{\left|h_{SD}\right|}^{2}\right]/N_{M}.$$

Similarly, the average *SNR* of the wiretap channel is described as:

(5) $$\gamma_{W}=P\times E\left[{\left|h_{SE}\right|}^{2}\right]/N_{W}.$$

The secrecy capacity in this model is defined as $C_{S}=C_{M}-C_{W}$:

(6) $$C_{S}=\{\begin{array}{l} \log\left(1+\frac{{\left|h_{SD}\right|}^{2}P}{N_{M}}\right)-\log\left(1+\frac{{\left|h_{SE}\right|}^{2}P}{N_{W}}\right)\\ 0 \end{array}\;\begin{array}{c} ,\;if\;\gamma_{M}>\gamma_{W}\\ ,\;if\;\gamma_{m}\leq \gamma_{W} \end{array}$$

We denote the instantaneous *SNR* of the main channel and wiretap channel by ${\gamma_{M}}^{\prime }$ and ${\gamma_{W}}^{\prime }$, [25]:

(7) $${\gamma_{M}}^{\prime }=P\times E\left[{\left|h_{SD}\left(i\right)\right|}^{2}\right]/N_{M},$$

(8) $${\gamma_{W}}^{\prime }=P\times E\left[{\left|h_{SD}\left(i\right)\right|}^{2}\right]/N_{W},$$

from which we can see that γ (regardless of the footnote in different channels) is exponentially distributed as $\gamma \propto {\left|h\right|}^{2}$. Then:

(9) $$p\left(\gamma \right)=\frac{1}{\bar{\gamma }}exp\left(-\frac{\gamma }{\bar{\gamma }}\right),$$

and the probability of $C_{S}>0$ (denoted by $P\left(C_{S}>0\right)$) is calculated in [8], specifically:

(10) $$P\left(C_{S}>0\right)=\int_{0}^{\infty }\int_{0}^{\gamma_{M}}p\left(\gamma_{M}\right)p\left(\gamma_{W}\right)d\gamma_{W}d\gamma_{M}=\frac{\gamma_{M}}{\gamma_{M}+\gamma_{W}}.$$

Then, we derive $P(C_{S}>0)$ in relay transmission model for different relay behaviors:

(1) $P(C_{S}>0)$ under cooperative behavior: The relay cooperatively decodes and forwards the received signal to D (or E). The destination combines the received signals from *S* and *R* by maximum ratio combining (MRC). The main channel consists of the S-R-D path and S-D path, and the wiretap channel includes the S-E and S-R-E path. $\gamma_{M}=\gamma_{SRD}+\gamma_{SD}$ is the SNR of the main channel, and $\gamma_{W}=\gamma_{SRE}+\gamma_{SE}$ is the *SNR* of the eavesdropper channel. Therefore, according to (10), the $P(C_{S}>0)$ is given by:
  (11) $$P_{C}\left(C_{S}>0\right)=\frac{\gamma_{SRD}+\gamma_{SD}}{\gamma_{SRD}+\gamma_{SD}+\gamma_{SRE}+\gamma_{SE}},$$
  where $\gamma_{SD}$ is the average *SNR* of *S* to *D* channel, $\gamma_{SRD}$ is the average *SNR* of the main channel which consists of *S* to *R*, and *R* to *D* path. The average *SNR* of the *S* to *E* path is denoted as $\gamma_{SE}$, and $\gamma_{SRE}$ is the average *SNR* of the wiretap channel which consists of *S* to *R*, and *R* to *E* path.
(2) $P(C_{S}>0)$ under selfish behavior: If the relay is selfish with the probability $P_{off}$, then, $\gamma_{M}=\gamma_{SD}$ and $\gamma_{W}=\gamma_{\mathrm{SE}}$, while the relay uses cooperative behavior with the probability ($1-P_{off}$). Then, $P(C_{S}>0)$ is given by:
  (12) $$P_{S}\left(C_{S}>0\right)=\left(1-P_{off}\right)\times \frac{\gamma_{SRD}+\gamma_{SD}}{\gamma_{SRD}+\gamma_{SD}+\gamma_{SRE}+\gamma_{SE}}+P_{off}\times \frac{\gamma_{SD}}{\gamma_{SD}+\gamma_{SE}}.$$
(3) $P(C_{S}>0)$ under malicious behavior: The received signal from the malicious relay at *D* and E is considered as a noise. $\gamma_{M}=\gamma_{SD}/\left(1+\gamma_{RD}\right)$ and $\gamma_{W}=\gamma_{SE}\left(1+\gamma_{RE}\right)$ , *P*(*Cs* > 0) is given by:
  (13) $$P_{M}\left(C_{S}>0\right)=\frac{\gamma_{SD}\left(1+\gamma_{RE}\right)}{\gamma_{SD}\left(1+\gamma_{RE}\right)+\gamma_{SE}\left(1+\gamma_{RD}\right)}.$$

### 2.4. Equivalent Signal-To-Noise Ratio from Source to the Destination

In theoretical analyses, most of the existing work considers the forward process as completely ideal in the DF strategy [10,11,24], in which the relay correctly decodes and forwards the received messages. However, A few researchers consider this a non-ideal scenario. Due to possible errors at the relay, the S-R-D channel is clearly non-linear and non-Gaussian. However, one can think of the BER in S-R-D channel as the error probability at the receiver of an equivalent one-hop AWGN link. The BER of the two-hop S-R-D channel is given by [26]:

(14) $$P_{SRD}=\left(1-P_{SR}\right)\times P_{RD}+\left(1-P_{RD}\right)\times P_{SR}$$

where $P_{SR}$ and $P_{RD}\;$ are the BER at source to relay (S-R) hop and relay to destination (R-D) hops, respectively. The equivalent SNR from the measured BER is applied to describe this non-ideal DF transmission scenario. Then the equivalent SNR is obtained.

## 3. Experimental Setup

**Figure 3:**
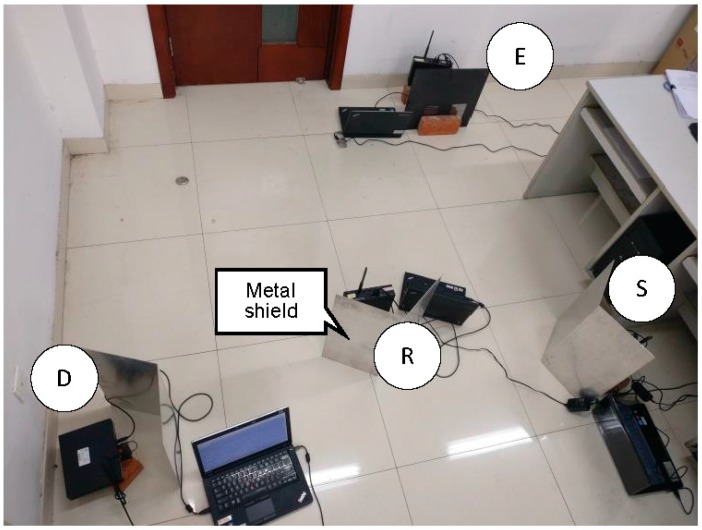
Experimental setup.

Software defined radio (SDR) is a rapidly emerging concept in wireless communication system. The main SDR involves using GNU Radio and universal software radio peripheral (USRP) in an Ubuntu system [18]. The experimental setup is shown in Figure 3, where each node is equipped with a laptop running GNU Radio connected to a USRP in the experiment. 

**Figure 4:**
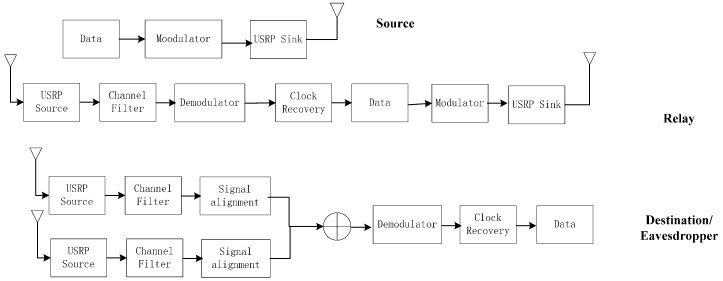
Signal processing at each node.

Metal shields are placed between nodes to help attenuate any line-of-sight path. The distance between every two points of S, R and D is 2 m. The noise power is measured about −60 dBm. The source power is fixed on −39 dBm and relay power changes from −51 dBm to −39 dBm (all the power presented in dBm is measured from the experiment). Figure 4 illustrates the different steps of signal processing at the transmitter and relay designed by the authors.

### 3.1. Source Operations

We use a USRP sink in the GNU Radio platform to transmit a binary bit stream. Messages are first encoded in the laptop to baseband signals. Then the USRP sink broadcasts the signal at 2.45 GHz with GMSK. A Python program is used to process the signals. We transmit 12,144 bits each time and repeat every situation 25 times to exclude an accident case. The signal’s power is defined in the software part by limiting the signal amplitude to a certain voltage.

### 3.2. Relay Operations

In this paper, we experiment on different relay strategy, forward power, and locations to discuss the influence on secrecy capacity. In general, the main task of a relay is to receive the signal from the source, decode and forward them to the destination. After received desired signal, it saves the complex signal for further processing and finally transmit processed signal in a certain power.

### 3.3. Data Receiving and Processing

The USRP both in destination and eavesdropper node operates in the same way. They monitor at the frequency of 2.45 GHz and save received complex signals to the laptop. Thanks to the extensive GNU Radio platform and the stable USRP hardware, we have little worries about how to generate a baseband bit stream and shifting it to a high frequency is much more easy and there is no carrier frequency offset problem compared to a traditional transmission device. The difficult part in the experiment however is processing the received data. The data are all saved in a complex signal form which can be read from MATLAB. We have to determine which part of the data is the desired signal and leave out the white noise. Besides, complex signals received at the destination and eavesdropper consists of the two parts received from the source and relay, respectively. Synchronization is required to combine these two parts and after synchronization, two different parts of the complex signal are combined using the maximum ratio combining (MRC) method.

## 4. The Physical Layer Security Performance of the Relay Transmission Model

**Figure 5:**
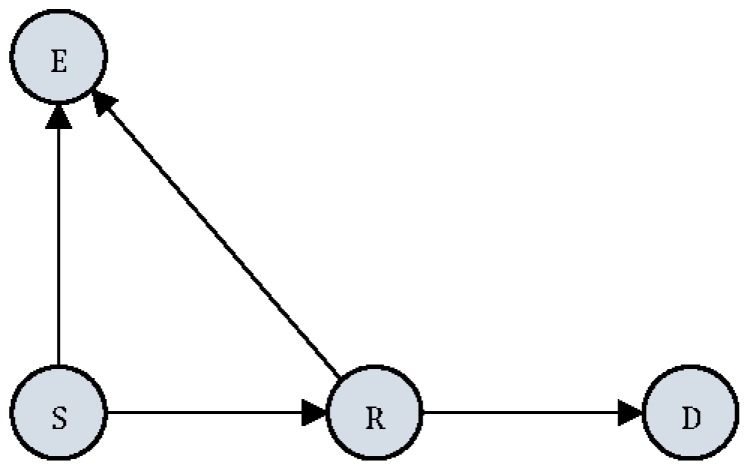
The system model.

Considering the model depicted in Figure 5, S, D and E are located at fixed two-dimensional normalized coordinates (0; 0), (1; 0) and (0; 1). R is located along a horizontal line with S and D. We execute the experiments and simulate the theoretical formulas according to Equations (11)–(13) for comparison.

### 4.1. The Influence of the Relay Power for P(Cs > 0)

#### 4.1.1. Simulation Results with Various Relay Power

Figure 5 is a typical cooperative relay communication model adopted from [7], with source, destination and eavesdropper nodes fixed and relay node’s location changing between the source and destination. The difference in this paper is that the relay has different relay behaviors. We simulate the $P(C_{S}>0)$ verses $P_{r}/P_{s}$ under different relay behaviors, where $P_{r}$ and $P_{s}$ are the power in relay and source, respectively, and the SNR of the source to destination (S-D) channel is 5dB. The path loss in the simulation is $1/d^{2}$, where d is the distance between two nodes.

**Figure 6:**
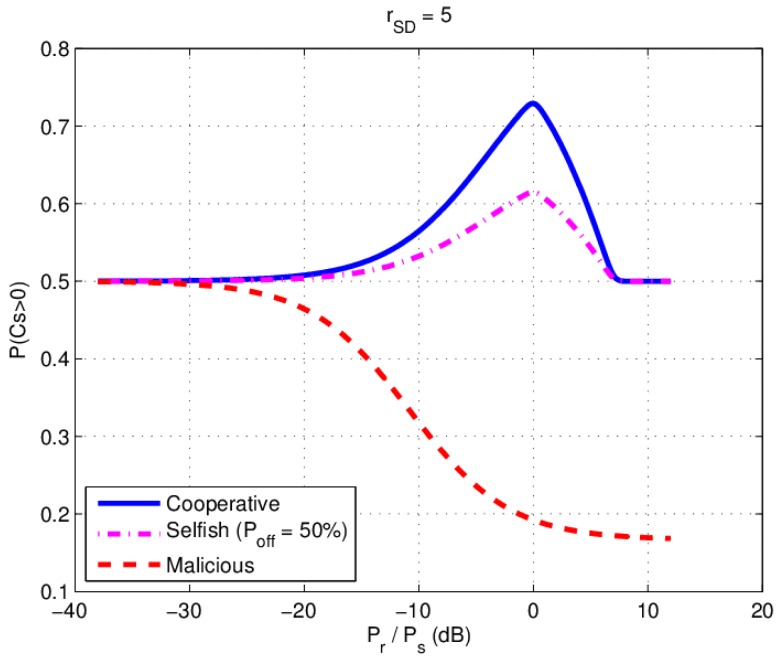
Simulation results:$\;P(C_{S}>0)$ versus $P_{r}/P_{S}$ under different behavior.

The relay’s normalized location is fixed at (0.5; 0) (the middle of S and D) and source power $P_{S}$ is fixed at −40 dBm. Relay power $P_{r}$ is changed from −70 dBm to −20 dBm. We simulate the $P(C_{S}>0)$ under three different relay behavior according to (11), (12) ($P_{off}=50\%$) and (13) as shown in Figure 6. We find that there exists an optimal relay power $P_{r}=P_{s}$, where $P(C_{S}>0)$ would reach a peak value in cooperative and selfish scenario. When the relay power $P_{r}$ increases beyond $P_{s}$, the$\;P(C_{S}>0)$ decreases. We also notice that increasing the malicious relay power always decreases the $P(C_{S}>0)$.

#### 4.1.2. Experimental Results with Various Relay Power

In the experiment, S, R, D and E are located at fixed two-dimensional coordinates (0; 0), (1.8 m; 0), (3.6 m; 0) and (0; 3.6 m), respectively. In both D and E node, BER of both main channel and wiretap channel are obtained by combining the complex signal from S and R using maximum-ratio-combining (MRC), which a weighted superposition of the source and relay signals arriving at the destination are collected with the maximum available diversity order. We calculate $P(C_{S}>0)$ by SNR according to (11), (12) and (13). The relay power is changed about 3 dBm every step and the source power is fixed at −39 dBm. In malicious behavior case, pseudo random noise is forwarded by the relay, in which there are 5972 error bits in all 12,144 bits.

**Figure 7:**
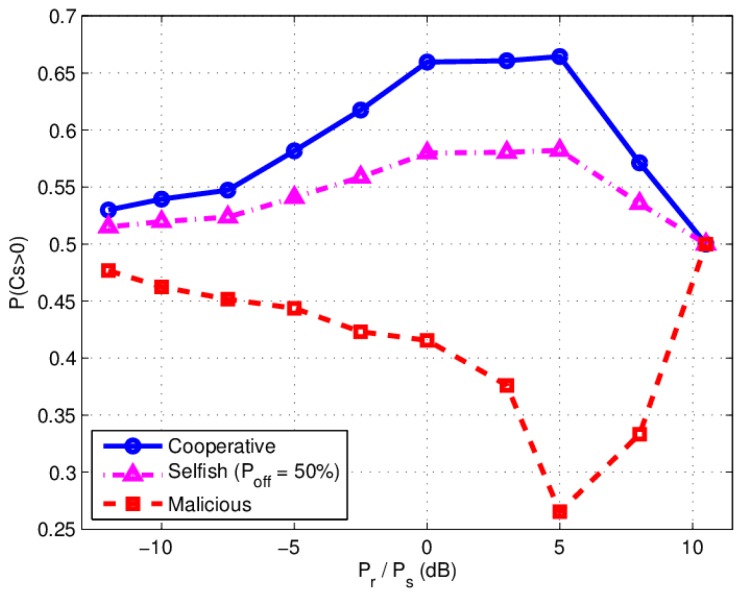
Experimental results: $P(C_{S}>0)$ versus $P_{r}/P_{s}$ under different behavior.

The experimental data of cooperative relay and malicious relay are shown in Table 2 and Table 3 respectively, where $P_{D}$ is the BER at the destination and $P_{E}$ is the BER at the eavesdropper. In Table 2, $\gamma_{M}=\gamma_{SRD}+\gamma_{SD}$ is the SNR of the main channel, and $\gamma_{W}=\gamma_{SRE}+\gamma_{SE}$ is the SNR of the eavesdropper channel. The last row of Table 2 is the calculated probability of $C_{S}>0$ (denoted by $P\left(C_{S}>0\right)$. In Table 3, $\gamma_{M}=\gamma_{SD}/\left(1+\gamma_{RD}\right)$ is the SNR of the main channel, and $\gamma_{W}=\gamma_{SE}\left(1+\gamma_{RE}\right)$ is the SNR of the eavesdropper channel. The graphic experimental results are shown in Figure 7.

In our experimental setup, the results from Figure 7 indicate that the optimal relay power value is larger than the sources power ($P_{r}/P_{s}=5\;\mathrm{dB}$), which means the relay need more power to achieve the optimal security performance in real non ideal DF environment. Therefore, generally speaking, in practice we can assume, the source to destination(S-D) channel is not ideal, the more relay power will be needed to achieve the same results as that in the ideal S-D channel.

Moreover, in malicious relay behavior case, $P(C_{S}>0)$ increases when $P_{r}/P_{s}$ is beyond 5 dB, which is different to theoretical simulation result. It indicates that when the malicious relay forwards noise power high enough, the eavesdropper channel will be deteriorated more heavily than the main channel. As for the influence of relay power, it can be found that there exists a maximum value of $P(C_{S}>0)$ for different relay powers.

### 4.2. Influence of Relay Location for P(C_S_ > 0)

#### 4.2.1. Simulation Results with Various Relay Location

**Figure 8:**
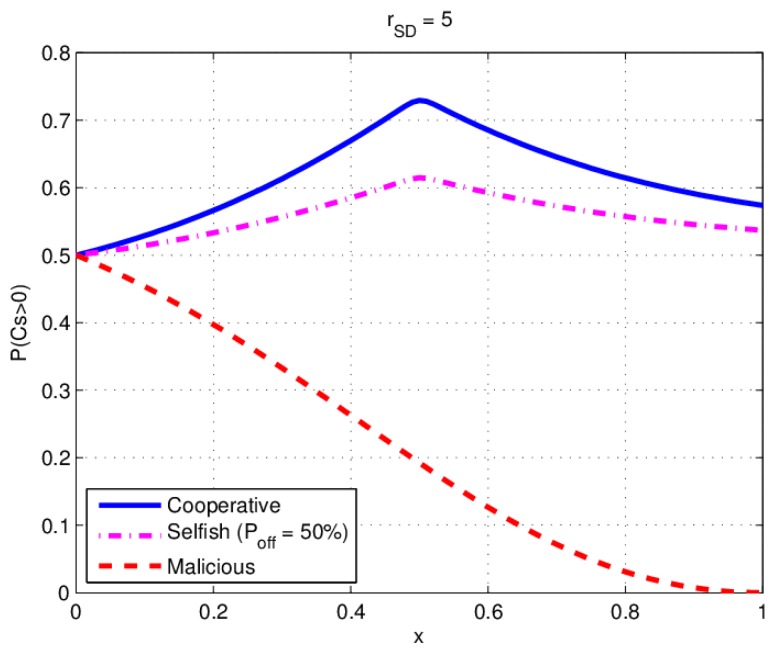
Simulation results: $P(C_{S}>0)$ versus relay location under various behaviors.

We assume $P_{r}$ equals $P_{s}$, and $x\left(x=d_{SR}/d_{SD}\right)$ is the normalized distance from *S* to *R*. Let x change from 0 to 1. The simulation result of $P(C_{S}>0)$ is shown in Figure 8. There exists an optimal relay location where $P(C_{S}>0)$ would reach a peak value in both cooperative and selfish behavior of a relay.

In the simulation $\gamma_{SD}=5$ is the SNR of S to D channel, the optimal location is in the middle of source and destination ($x=d_{SR}/d_{SD}=0.5$). $P(C_{S}>0)$ will decreases quickly when R moves towards D with a malicious behavior relay.

#### 4.2.2. Experimental Results with Various Relay Location

**Figure 9:**
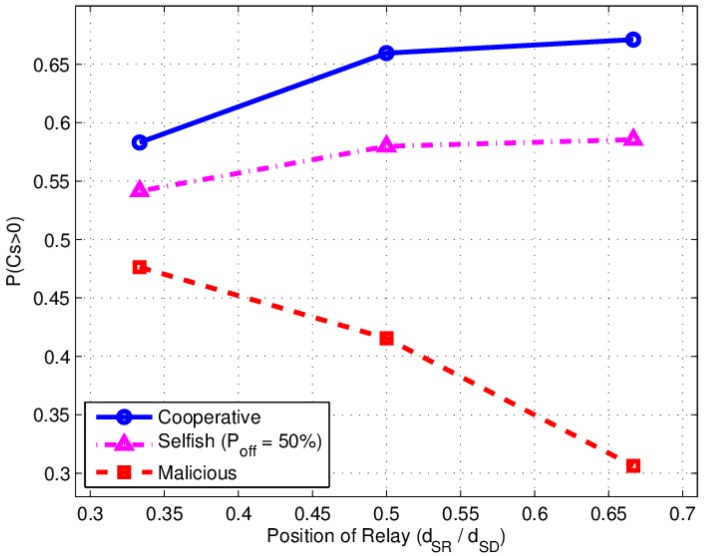
Experimental results: $P(C_{S}>0)$ versus relay location under various behavior.

In this experiment, the transmit power of S and R both are fixed at −39 dBm. S, D and E are located at fixed two-dimensional coordinates (0, 0), (3.6 m, 0) and (0, 3.6 m), respectively. The coordinate of R changes from (1.2 m, 0), (1.8 m, 0) to (2.4 m, 0). The experimental results table are shown in Table 4 and Table 5 respectively. The graphic experimental result is shown in Figure 9.

It can be seen that the experimental $P(C_{S}>0)$ value in Figure 9 is different from the theoretical curves in Figure 8. The optimal location of the relay is a nearer the destination in experiment with non ideal DF case, whereas in theoretical ideal DF analysis, the location is in the middle of the Source to destination link. That phenomenon is also appeared in selfish relay case. For malicious behavior, the near destination malicious relay deteriorates the main channel seriously, but it is not as serious as that in theoretical simulation.

## 5. Conclusions

In this paper, we implement several experiments to investigate the physical layer security of a relay transmission model with different relay behaviors. Experimental results are compared with a theoretical analysis. We find a gap between the ideal theoretical simulations and the non-ideal real experiments:

(1) The relays need more power to achieve the maximum-security performance in a real environment. In the experiment, the optimal relay power is larger than the source power in both cooperative and selfish behavior relay scenarios because of the non-ideal source to destination (S-D) channel;
(2) In the malicious behavior relay case, the experimental value of $P(C_{S}>0)$ is different from the ideal theoretical simulation result when the relay power is larger than the critical power ($P_{r}/P_{S}=5\;\mathrm{dB}$), which indicates that when s malicious relay forwards high enough noise power, the eavesdropper channel is deteriorated more seriously than the main channel;
(3) Optimal relay location in both cooperative and selfish behavior of relay is in the middle of source and destination ($d_{SR}/d_{SD}=0.5$) in the ideal theoretical simulation. However, in the experiment, the optimal relay location is nearer to the destination because of the non-ideal source to relay and to destination (S-R-D) channel.

## Figures and Tables

**Table 1:** Symbols Description.

$h_{SD}$	the channel fading coefficients from S to D (source to destination)
$h_{SR}$	the channel fading coefficients from S to R (source to relay)
$h_{RD}$	the channel fading coefficients from R to D (relay to destination)
$P_{off}$	the probability in the Off-State
$P(C_{S}>0)$	the probability of Non-zero secrecy capacity
$\gamma_{M}$	the average SNR of the main channel
$\gamma_{W}$	the average SNR of the wiretap channel
$\gamma_{SD}$	the average SNR of the S to D channel (source to destination)
$\gamma_{SRD}$	the average SNR of the main channel which consists of channel from S to R and R to D
$\gamma_{SE}$	the average SNR of the S to E path
$\gamma_{SRE}$	the average SNR of the wiretap channel which consists of channel from S to R, and R to E
$P_{SR}$	the BER at S-R hop
$P_{RD}$	the BER at R-D hop
$P_{SRD}$	the BER of the two-hop S-R-D channel

**Table 2:** Experimental results with different transmit power under cooperative relay.

Transmit Power (dBm)	−51	−49	−46.5	−44	−41.5	−39	−36	−34	−31	−28.5
$P_{D}$	0.3383	0.2472	0.1873	0.113	0.061	0.0196	0.0026	0.0002	0.0001	0.0000
$\gamma_{M}$	0.0974	0.2614	0.4415	0.825	1.3386	2.3814	4.3727	7.1942	8.1577	∞
$P_{E}$	0.3472	0.2639	0.2096	0.152	0.1118	0.0692	0.0226	0.0054	0.0005	0.0000
$\gamma_{W}$	0.0864	0.2231	0.3652	0.593	0.8293	1.2289	2.2451	3.6327	6.1224	∞
$P(C_{S}>0)$	0.5299	0.5394	0.5473	0.582	0.6175	0.6596	0.6608	0.6645	0.5713	0.5

**Table 3:** Experimental results with different transmit power under malicious relay.

Transmit Power (dBm)	−51	−49	−46.5	−44	−41.5	−39	−36	−34	−31	−28.5
$P_{D}$	0.3670	0.3810	0.4017	0.4198	0.4358	0.4459	0.4635	0.4811	0.4878	0.4918
$\gamma_{M}$	0.0647	0.0513	0.0343	0.0229	0.0146	0.0104	0.0047	0.0013	0.0005	0.0002
$P_{E}$	0.3609	0.3725	0.3902	0.4104	0.4251	0.4359	0.4530	0.4685	0.4847	0.4918
$\gamma_{W}$	0.0710	0.0592	0.0416	0.0287	0.0200	0.0146	0.0078	0.0035	0.0010	0.0002
$P(C_{S}>0)$	0.4767	0.4624	0.4519	0.4438	0.4231	0.4155	0.3761	0.2652	0.3333	0.5

**Table 4:** Experimental results with various relay locations of cooperative behavior.

Relay Location	Left (1.2 m, 0)	Middle (1.8 m, 0)	Right (2.4 m, 0)
$P_{D}$	327.08	237.84	383.27
$\gamma_{M}$	2.081	2.3814	1.9336
$P_{E}$	625.71	840.78	1174.15
$\gamma_{W}$	1.488	1.2289	0.9472
$P(C_{S}>0)$	0.5831	0.6596	0.6712

**Table 5:** Experimental results with different relay location of malicious behavior.

Relay Location	Left (1.2 m, 0)	Middle (1.8 m, 0)	Right (2.4 m, 0)
$P_{D}$	0.441	0.4459	0.4535
$\gamma_{M}$	0.0123	0.0104	0.0076
$P_{E}$	0.4382	0.4359	0.4302
$\gamma_{W}$	0.0135	0.0146	0.0173
$P(C_{S}>0)$	0.4763	0.4155	0.3063

## References

[1] Schneier B. Description of a new variable-length key, 64-bit block cipher (Blowfish). Proceedings of the Fast Software Encryption: 7th International Workshop, FSE 2000.

[2] Bruce S. (1996). Applied Cryptography: Protocols, Algorithms, and Source Code in C.

[3] Yang N., Wang L., Geraci G., Elkashlan M., Yuan J., Di Renzo M. (2015). Safeguarding 5G wireless communication networks using physical layer security. IEEE Commun. Mag..

[4] Wang H.M., Zheng T.X. (2016). Physical Layer Security in Heterogeneous Cellular Network. Physical Layer Security in Random Cellular Networks.

[5] Wyner A.D. (1975). The Wire-Tap Channel. Bell Labs Techn. J..

[6] Shannon C.E. (1949). Communication theory of secrecy systems. Bell Labs Techn. J..

[7] Lai L., El Gamal H. (2008). The relay–eavesdropper channel: Cooperation for secrecy. IEEE Trans. Inf. Theory.

[8] Dong L., Han Z., Petropulu A.P., Poor H.V. (2010). Improving wireless physical layer security via cooperating relays. IEEE Trans. Signal Process..

[9] Bassily R., Ulukus S. (2013). Deaf cooperation and relay selection strategies for secure communication in multiple relay networks. IEEE Trans. Signal Process..

[10] Bassily R., Ulukus S. (2012). Deaf cooperation for secrecy with multiple antennas at the helper. IEEE Trans. Inf. Forensics Secur..

[11] Bassily R., Ulukus S. (2012). Secure communication in multiple relay networks through decode-and-forward strategies. J. Commun. Netw..

[12] Chen X., Lei L., Zhang H., Yuen C. (2015). Large-scale MIMO relaying techniques for physical layer security: AF or DF?. IEEE Trans. Wirel. Commun..

[13] Chen X., Zhong C., Yuen C., Chen H.H. (2015). Multi-antenna relay aided wireless physical layer security. IEEE Commun. Mag..

[14] Li Q., Yang Y., Ma W.K., Lin M., Ge J., Lin J. (2015). Robust cooperative beamforming and artificial noise design for physical-layer secrecy in AF multi-antenna multi-relay networks. IEEE Trans. Signal Process..

[15] Zhang J., Yuen C., Wen C.K., Jin S., Wong K.K., Zhu H. (2016). Large system secrecy rate analysis for SWIPT MIMO wiretap channels. IEEE Trans. Inf. Forensics Secur..

[16] Salem A., Hamdi K.A., Rabie K.M. (2016). Physical layer security with RF energy harvesting in AF multi-antenna relaying networks. IEEE Trans. Commun..

[17] Liu Y., Chen H.H., Wang L. (2016). Physical layer security for next generation wireless networks: Theories, Technologies, and Challenges. IEEE Commun. Surv. Tutor..

[18] Xiang H., Yener A. Secrecy and reliable Byzantine detection in a Gaussian untrusted two-hop link. Proceedings of the 2010 IEEE Information Theory Workshop on Information Theory (ITW 2010).

[19] He X., Yener A. (2010). Cooperation with an untrusted relay: A secrecy perspective. IEEE Trans. Inf. Theory.

[20] Hou L., Fu X. (2014). Physical layer security with dynamic behaviour cooperator based on coalitional game. IET Commun..

[21] Fu X., Li L., Zong Q. (2015). Bayesian Coalitional Game in Physical Layer Security. Wirel. Pers. Commun..

[22] Chrysikos T., Dagiuklas T., Kotsopoulos S. (2011). Wireless information-theoretic security in an outdoor topology with obstacles: Theoretical analysis and experimental measurements. EURASIP J. Wirel. Commun. Netw..

[23] Mitola J. (1995). The software radio architecture. IEEE Commun. Mag..

[24] Zhang R., Comaniciu C., Poor H.V. Outage capacity and partial secrecy for energy efficient physical layer security in Gaussian fading channels. Proceedings of the 2013 16th International Symposium on Wireless Personal Multimedia Communications (WPMC).

[25] Barros J., Rodrigues M.R. Secrecy capacity of wireless channels. Proceedings of the 2006 IEEE International Symposium on Information Theory.

[26] Wang T., Cano A., Giannakis G.B., Laneman J.N. (2007). High-performance cooperative demodulation with decode-and-forward relays. IEEE Trans. Commun..

